# Epigenetic links between paternal age, reproduction, and offspring health: a focus on sncRNAs

**DOI:** 10.1530/RAF-25-0093

**Published:** 2025-12-08

**Authors:** Naledi Matenge, Jonathan P Evans, Jacqueline Batley, Renée C Firman

**Affiliations:** Centre for Evolutionary Biology, School of Biological Sciences, University of Western Australia, Crawley, Western Australia, Australia

**Keywords:** sperm, epigenetics, environmental effects, andrology

## Abstract

**Abstract:**

As men age, their reproductive capacity changes. Although males do not have a defined ‘biological clock’, evidence suggests that paternal age plays a pivotal role in male reproduction and offspring health. Epigenetic mechanisms, which influence how genes are expressed in all cells, are important in gametogenesis and can be influenced by endogenous factors such as diet, toxin exposure, stress, and age. Hence, an altered paternal environment can generate epigenetic modifications that can be passed onto the offspring via the ejaculate. In humans, more couples in developed countries are delaying parenthood and are relying on assisted reproductive techniques. Although research has focused on maternal age, growing evidence shows that advanced paternal age (APA) can influence the epigenetic pathways in embryos and offspring. There are several known epigenetic mechanisms that may link APA to downstream fitness effects in offspring: DNA methylation, histone post-translational modifications, chromatin remodelling, and small noncoding RNA (sncRNA) expression. Sperm RNAs were once considered residual products of sperm maturation. Now, small RNAs have emerged as key players in male fertility. sncRNA expression tightly regulates the sperm cell cycle and maturation, and embryo development. Despite their importance in epigenetic inheritance, the relationship between paternal age, sncRNA expression, and reproductive outcomes is unclear. Given the role of sncRNA expression in gametogenesis and embryogenesis, understanding how age influences sncRNA expression could provide insights into the underlying factors of failed natural and assisted fertility. This review explores the links between paternal age, sperm epigenetics, reproduction, and offspring health, with a focus on sncRNA expression.

**Lay summary:**

Unlike women, men do not have a clear biological clock. However, research shows that a man’s age can affect his fertility and the health of his children. As men get older, the way genes are turned on and off in sperm changes and these changes can be passed on to their children. Lifestyle factors such as diet, stress, and exposure to toxins can affect how genes are controlled. Much research has focused on the effects of maternal age, but there is growing evidence that advanced paternal age (APA) can influence fertility and embryo development. A key area of interest is small sperm molecules that influence early development. Once thought to be mere by-products of sperm development, these molecules are now known to play crucial roles in sperm function and embryo development. This review examines how a father’s age affects these small sperm molecules and their role in fertility and offspring health.

## Background

Male factor infertility affects approximately 50% of infertile human couples. According to the European Association of Urology Guideline for Sexual and Reproductive Health, 20% of infertile couples suffer from pure male factor infertility (Salonia *et al.* 2025). Although there is no universally defined age at which males are considered to be ‘advanced’, the American College of Medical Genetics defines this as age 40 at the time of conception (see Toriello & Meck (2008)). APA-associated changes in natural fertility are typically attributed to physiological changes that affect the male reproductive system. For example, studies have shown links between APA and dysregulated reproductive hormone levels and decreased sperm quality (Ashapkin *et al.* 2023). Reduced semen quality is often associated with increased sperm DNA damage (reviewed in Ashapkin *et al.* (2023)). Clinical outcomes, including spontaneous miscarriage rates, live birth rates (du Fossé *et al.* 2020), and embryological outcomes of fertility techniques, such as *in vitro* fertilisation (IVF) or intracytoplasmic sperm injection (ICSI), are also affected by APA (Van Opstal *et al.* 2021, Mazzilli *et al.* 2025). A systematic review and meta-analysis of ten studies conducted in humans found that paternal age is associated with spontaneous miscarriage rates (du Fossé *et al.* 2020). Furthermore, APA can negatively influence human embryo development and quality; the former can be identified by the number of blastomeres present on days 2 and 3 of embryo culture (Van Opstal *et al.* 2021, Mazzilli *et al.* 2025).

There is now substantial evidence that APA is linked to overall offspring health. For example, research dating back to 1958 has shown a link between APA and schizophrenia (Johanson 1958). Nearly seven decades later, extensive data from empirical studies using animals and correlative human research further implicate APA in neuropsychiatric disorders, including bipolar disorder, autism, and obsessive-compulsive disorder (Sharma *et al.* 2015, Fang *et al.* 2020, Halvaei *et al.* 2020, Raposo-Amaral *et al.* 2020, Gourinat *et al.* 2023). APA has been implicated in several perinatal disorders, which include congenital anomalies and aneuploidies. Congenital anomalies are conditions that occur prenatally and manifest at birth, likely impacting an offspring’s health, development, and/or survival (DeSilva *et al.* 2016). Such anomalies can affect several organ systems, such as the cardiovascular, musculoskeletal, digestive, and neurological systems in offspring (Nybo Andersen & Urhoj 2017, Fang *et al.* 2020). Offspring from older fathers are more susceptible to congenital disorders, such as Apert syndrome, Klinefelter syndrome, and childhood cancers (Fang *et al.* 2020, Halvaei *et al.* 2020, Raposo-Amaral *et al.* 2020). A large, population-based retrospective study on the effects of paternal age on structural birth defects was conducted and found that older fathers, after adjusting for maternal age, had babies born with various congenital malformations (Grewal *et al.* 2012). Examples of these malformations include chondrodystrophy, a type of dysplasia which causes disproportionate dwarfism (Schwartz & Domowicz 2004), and other limb anomalies affecting the pelvic girdles and lower limbs (Grewal *et al.* 2012). The aetiology of the association between APA and offspring health is not fully understood, although it is likely caused by several factors such as genetic *de novo* mutations or epigenetic alterations (Aguiar Soares *et al.* 2025).

Epigenetics is the study of heritable modifications in gene expression that do not change the underlying DNA sequence of an organism but instead are attributable to factors that alter the chromatin structure (Cheuquemán & Maldonado 2021). There are several known epigenetic mechanisms, including DNA methylation/demethylation, post-translational histone modifications, remodelling of nucleosomes (higher-order chromatin formation), and small noncoding RNAs (sncRNAs) (Cheuquemán & Maldonado 2021). Mature sperm cells carry a repertoire of essential RNAs, which includes small RNAs that are less than 200 nucleotides in length (Chan *et al.* 2022). These RNAs include small noncoding RNAs (sncRNAs), which are transferred to maturing sperm cells via specialised extracellular vesicles called epididymosomes (Belleannée *et al.* 2013). Sperm cells acquire different sncRNAs as they mature along the male reproductive tract, between the testis and cauda (distal) epididymis (Belleannée *et al.* 2013, Sharma *et al.* 2018). Data suggest a soma-to-germline transfer of sncRNAs to maturing sperm cells via epididymosomes. The caput (proximal) region of the epididymis, specifically the epithelial cells, synthesises the microRNAs (miRNAs) present in maturing sperm. In addition, similar RNA payloads have been found in purified epididymosomes relative to those gained during sperm maturation (Sharma *et al.* 2018). Mouse embryos developed from sperm collected from the caput epididymis failed to implant efficiently, but when injected with caudal-specific small RNAs, preimplantation embryo developmental rates were rescued (Conine *et al.* 2018, Trigg & Conine 2024), and the post-implantation embryo lethality phenotype was reduced (Sharma *et al.* 2018). A similar study found that sperm with a partially depleted miRNA and endogenous small interfering RNA repertoire produced embryos with abnormal preimplantation development. This phenotype was then rescued when wild-type total and small sperm RNAs were microinjected into eggs fertilised by abnormal sperm (Yuan *et al.* 2015). Researchers have even demonstrated that testicular sperm with depleted miRNAs are able to restore 72% of lost miRNAs upon epididymal transport (Trigg & Conine 2024). Despite demonstrating a causal link between sperm RNAs and altered embryo development, there are still some unanswered questions that need to be addressed, especially if we are to fully understand the paternal age effects on sperm sncRNAs, and subsequent effects on offspring health. First, analysis of the sncRNAs involved in aberrant embryo phenotypes is often biased towards a few RNA groups, namely miRNAs and tsRNAs. These sncRNAs are exclusively selected for microinjection, and the resulting phenotype is attributed to those chosen sncRNAs without considering others (which are likely in smaller concentrations). Second, the mechanistic action of sncRNAs on embryo development is not fully understood. When exploring these molecular mechanisms, several considerations need to be made. These include establishing the localisation of sperm RNAs (head vs tail), their half-life, and their interaction potential in the zygote or embryo. Together, these factors highlight the gap in our knowledge on the effect of sperm small RNAs in pre- and post-implantation development (Chen 2022). However, studies conducted thus far demonstrate the critical role of sperm cell sncRNAs at the time of fertilisation and emphasise the importance of future studies on the topic (Chen 2022).

Despite it being established that APA is linked to an increased incidence of diseases and abnormalities in offspring, researchers have only recently begun to identify the mechanisms by which paternal age effects are transmitted. One of the earliest studies by Jenkins *et al.* (2013) determined that global sperm DNA methylation patterns increase with age. However, the effect of APA on sperm sncRNAs has not been studied in detail. Given that this research topic is in its infancy, there is one notable review that collates data collected to date by Ashapkin *et al.* (2023). This review provides a prospective evaluation of the influence of paternal age on a range of epigenetic mechanisms, including DNA methylation, post-translational histone modifications, and sperm sncRNAs, and discusses their potential effect on embryological and clinical reproductive outcomes, as well as offspring health.

## Paternal age and sperm epigenetics

Epigenetic mechanisms alter gene expression not by changing the underlying DNA sequence, but by modifying the chromatin structure (Kubota *et al.* 2012, Jenkins *et al.* 2013), and these changes can be passed between generations (Singh *et al.* 2023). DNA methylation is the most studied and well-characterised (Kubota *et al.* 2012, Jenkins *et al.* 2013) mechanism of gene silencing. The following section describes studies that identified a link between paternal age, DNA methylation, and histone modifications.

### DNA methylation

Mammalian gametes and embryos are subjected to epigenetic remodelling via DNA methylation/demethylation. In sperm cells, about 90% of the genome is evenly methylated, except for specific CpG islands. CpG islands are palindromic stretches (300 to 3,000 base pairs) of DNA made up of cytosine (C) and guanine (G) nucleotides (Singh *et al.* 2023). During DNA methylation, DNA methyltransferases (DNMTs) mediate the addition of a methyl group to the 5-position of cytosine, forming 5-methylcytosine (5 mC). Conversely, ten-eleven translocation (TET) enzymes facilitate demethylation of 5 mC groups (Wang *et al.* 2021).

Upon fertilisation, parental genomes within zygotes undergo demethylation, which erases most DNA methylation patterns carried from the maternal and paternal genomes. The paternal genome is rapidly demethylated, while the maternal genome is passively and gradually demethylated. Once a mouse embryo reaches the blastocyst stage (E4.5), DNA methylation levels are at their lowest (Singh *et al.* 2023). Preimplantation embryos contain hundreds of genes that resist DNA demethylation. These genes are imprinting genes and are monoallelically expressed in a parent-of-origin-specific manner (Sleutels & Barlow 2002, Denomme *et al.* 2020). Few studies have shown how paternal effects influence genomic imprinting and the impact this has on offspring (Zheng *et al.* 2021, Zhang *et al.* 2023; reviewed in Denomme *et al.* (2020)). In mice and humans, progression from the blastocyst stage to the gastrula stage is marked by *de novo* DNA methylation via the DNMT3a and DNMT3b enzymes. The enzyme DNMT1 maintains methylation levels in somatic cells, while TET enzymes induce global demethylation in primordial germ cells. Methylation in sperm cells, through the action of DNMT3a and DNMT3b, slowly increases until fully restored before birth (Singh *et al.* 2023).

Paternal age was first linked to DNA methylation patterns in 1987 by Wilson *et al.*, who reported that age affected DNA methylation loss in tissues of two rodent models (Wilson *et al.* 1987). Since then, multi-omics techniques have advanced to the extent that the methylome and transcriptome of a single cell can be analysed so that DNA methylation variation and gene expression can occur simultaneously (Hu *et al.* 2016).

As investigative epigenetic tools continue to evolve, our understanding of the effects of paternal age on sperm DNA methylation patterns is advancing. Given these dynamic developments, here we focus on research from the last 5 years to ensure that we capture findings that reflect the most current insights. This research highlights the effect of paternal age on sperm DNA methylation profiles and the associated changes in gene expression (Oluwayiose *et al.* 2021, Yoshizaki *et al.* 2021, Bernhardt *et al.* 2023, Potabattula *et al.* 2023). Consequently, the biological systems associated with those differentially expressed genes are also affected by paternal age.

Although research has found that APA-related changes can occur in the sperm methylome, some studies have linked paternal age to overall increased hypermethylation of differentially methylated regions (DMRs), while others have associated it with increased hypomethylation of DMRs (Oluwayiose *et al.* 2021, Yoshizaki *et al.* 2021, Bernhardt *et al.* 2023, Potabattula *et al.* 2023). These differences could be attributed to i) sample size (including variation and low numbers), ii) differences in the definitions of what constitutes a DMR, iii) sample composition and purification methods, and/or iv) a range of biological factors (reviewed in Kotková & Drábek (2023)). It is difficult to control for all these factors in one investigation; however, collectively they emphasise that paternal age is an influential factor in DNA methylation profiles of DMRs.

Yoshizaki *et al.* (2021) found that paternal age was primarily associated with hypomethylation of DMRs. Gene ontology (GO) analyses of hypomethylated DMRs revealed significant enrichment of genes involved in neurological functions and structures. The genes related to hypomethylated DMRs were found in gene lists linked to ‘abnormal nervous system physiology,’ ‘nervous system phenotype,’ and ‘abnormal long-term depression.’ A significantly enriched unique genomic sequence was found in the hypomethylated DMRs of sperm from aged mice (Yoshizaki *et al.* 2021). The sequence was deemed a potential binding motif for the transcription repressor REST/NRSF complex, which plays a pivotal role in embryonic brain development and has been linked to Down syndrome (Canzonetta *et al.* 2008, Yoshizaki *et al.* 2021, Jin *et al.* 2023). In addition, Yoshizaki *et al.* found that there was a significant reduction in the DNA methylation ratio of CpG sites of two autism-related genes, *Nav1* (*Scn2a*) and *Shank2*, in aged male mice (Yoshizaki *et al.* 2021).

There are over 800 genes associated with autism spectrum disorder (ASD) (Genovese & Butler 2023). The human *BEGAIN* gene encodes a brain-enriched guanylate kinase-associated protein, which is thought to promote postsynaptic neurotransmitter activity. Paternal age was found to have a significant negative correlation with *BEGAIN* methylation in sperm samples of normozoospermic men (Potabattula *et al.* 2023). Moreover, analysis of human foetal cord blood (FCB) revealed that paternal age was inversely proportional to *BEGAIN* methylation, specifically in male FCB samples. The analysis method used on FCB samples measured both maternal and paternal DNA molecules in the genomic DNA sample. To distinguish between parental alleles in the offspring genome, a bisulphite pyrosequencing genotyping assay that targets a genetic variant (rs7141087) in the *BEGAIN* promoter region was developed (Potabattula *et al.* 2023). Although a relationship between paternal age and paternal allele methylation in male FCB was observed, it was not statistically significant. Despite the lack of statistical significance, Potabattula *et al.* (2023) compared *BEGAIN* methylation levels in the peripheral blood of male participants with ASD with age- and sex-matched controls. A highly significant correlation between hypomethylation of the *BEGAIN* promoter region and the presentation of ASD was observed. This finding was used to extrapolate that APA is linked to ASD via hypomethylation of the *BEGAIN* promoter region (Potabattula *et al.* 2023). One should be cautious in making such a causal link, as other demographic factors of the autistic participants used were not stated, such as parental age at conception.

Bernhardt *et al.* (2023) found that there were more hypomethylated (74%) age-related DMRs (ageDMRs) compared to those that were hypermethylated (26%). Furthermore, according to the Genomic Regions Enrichment of Annotations Tool, hypomethylated ageDMRs were associated with mouse and human infertility phenotypes (Bernhardt *et al.* 2023). Hypermethylated ageDMRs were overrepresented in biological processes involving synapse formation and in abnormal behavioural human phenotypes. This contrasts with Yoshizaki *et al.*’s findings, which attributed abnormal neurodevelopment and behavioural phenotypes to hypomethylated ageDMRs (Yoshizaki *et al.* 2021). This discrepancy could be due to several differences in the methodologies used in the two studies, including the sequencing platforms used by each group, enrichment tools used, tissue types sampled, and the difference in ages of the participants/subjects. For example, Yoshizaki’s group (Yoshizaki *et al.* 2021) used post-bisulfite adaptor tagging (PBAT) to analyse ageDMRs in sperm and the forebrain of offspring sired by old (>12 months) and young (3 months) mice. On the other hand, Bernhardt *et al.* (2023) used reduced representation bisulfite sequencing (RRBS) to measure ageDMRs in men aged 25.8–50.4 years old. RRBS is biased to areas of the methylome that are rich in CpG islands; hence, the prevalence of hypermethylated ageDMRs may be inflated compared to data collected using PBAT (Soto *et al.* 2016). PBAT is less restrictive, adopting a whole-genome bisulphite sequencing approach that can detect CpG-poor regions at the single-cell level (Miura *et al.* 2012). An observational study on humans found that as male age increases, so did methylation of the majority (91%, *n* = 1,546) of targeted CpG sites. Following biological pathway analyses of ageDMRs, the top enriched pathways identified were those involved in embryonic development and neurodevelopment (Oluwayiose *et al.* 2021). This study, such as that of Yoshizaki *et al.* (2021), found that paternal age was linked to loss of methylation in genes involved in embryo development (Oluwayiose *et al.* 2021). However, this study attributes paternal age to an increase in methylation of behaviour-associated genes, such as the dopamine receptor D3.

Oluwayiose *et al.* (2021) found sperm ageDMRs associated with four genes: *DEFB126*, *PLCH2*, *TPI1P3,* and *DLGAP2*, which are potential mediators of male age effects on fertilisation rates and live birth rates in an assisted reproduction technique setting (Oluwayiose *et al.* 2021). This study highlighted two genes, *DEFB126* and *DLGAP2,* which assist with successful fertilisation and are linked to male infertility, respectively. Men with a homozygous *DEFB126* nucleotide frameshift deletion produce ejaculates that result in longer time to pregnancy compared with the general population (Oluwayiose *et al.* 2021). In addition, sperm with this deletion have an 84% reduction in penetration assays, which are used to determine how well sperm move in cervical mucus (Boroujeni *et al.* 2019, Oluwayiose *et al.* 2021). Genome-wide methylation patterns of semen samples were assessed, and it was determined that for every 5-year increase in male age, methylation of CpG sites increased by 0.2–11.7% (Oluwayiose *et al.* 2021).

Another study found that age and exposure to an environmental xenobiotic, 2,2′,4,4′-tetrabromodiphenyl ether (BDE-47), altered the sperm methylome in Wistar rats. In aged, unexposed rats, ageDMRs were hypermethylated and were enriched in terms related to embryo development, morphogenesis, and neuron and brain development. Interestingly, exposure to BDE-47 in young rats resulted in similar sperm genome methylation levels as those of sperm from aged control rats. Pilsner *et al.* (2021) were also able to demonstrate synergy between genomic DNA methylation and sncRNA profiles during ageing. They identified 1,052 genes that overlapped between miRNA gene targets and ageDMR-associated genes, and they were highly enriched for categories associated with embryo development, such as morphogenesis, brain development, and heart development (Pilsner *et al.* 2021).

Given these data, there is the potential for sperm DNA methylation profiles to be used as a predictive tool of sperm chronological age. A balance between biological and chronological age is an important factor in human fertility and fecundity (Maltoni *et al.* 2022). Chronological age refers to the time elapsed from birth to a given date. Biological age, on the other hand, reflects a combination of chronological age and additional factors that contribute to cellular damage, such as lifestyle and environmental exposures (Maltoni *et al.* 2022). Indeed, several studies found that ageDMRs can be used as biological clocks to predict fecundity and time to pregnancy. However, small sample sizes and the need for advanced machine-learning tools are limiting factors in these studies (Cao *et al.* 2020, Pilsner *et al.* 2022).

### Histone modification

Post-translational modifications (PTMs) of histone variants occur when histone-modifying enzymes covalently add or remove organic groups such as acetyl, methyl, phosphate, and ubiquitin (Chioccarelli *et al.* 2020). Interactions between PTMs on histone tails and DNA regulate chromatin structure, thereby influencing gene expression (Chioccarelli *et al.* 2020). Specific histone PTMs are essential for germ cell development, namely spermatogonial stem cell renewal, meiotic recombination, and nuclear condensation in spermatids (Chioccarelli *et al.* 2020). During the latter part of spermatogenesis, sperm cells undergo a wave of nuclear condensation, facilitated by the displacement of most histones for protamines, preserving only a small number of nucleosomes (histone octamer–chromatin subunit). These preserved regions are enriched with developmental gene promoters and imprinting gene loci (Hammoud *et al.* 2011, Chioccarelli *et al.* 2020). Research has shown that protamines are also subjected to PTMs but the molecular functions of these modifications are not clear. It is suggested that PTMs in protamines represent a ‘protamine code’ which is subjected to epigenetic inheritance (reviewed Arévalo *et al.* (2022)). Given this, we will only discuss data collected on how histone methylation marks are affected by paternal age.

To the best of our knowledge, there are only two studies, one conducted by Xie *et al.* (2018) and the other by Tatehana *et al.* (2020), which have shown a relationship between paternal age and histone modifications. Xie *et al.* (2018) discovered that 90% of the differential PTMs were located on a small region (∼1 Kbp) of chromosome 5 and observed a difference in PTMs with age, focussing on H3K4me3 and H3K27me3 retention. Importantly, the investigators found that the region consists of a family of spermatogenesis-related genes. These data, albeit correlative, show that paternal age may influence the reproductive capacity of sperm by altering modifications of histones located on chromosome 5 (Xie *et al.* 2018). Tatehana *et al.* (2020) compared histone modifications between young (3 months) and aged mice (12 months) and found that the immunohistochemistry (IHC) signal intensity, not localisation, of histone PTMs was altered. The authors found that sperm H3K9me3 and H3K4me2 intensity decreased, while H3K27me2/3 intensity increased with paternal age, which led to the suggestion that a decrease in H3K9me3 could cause aberrant meiotic sex chromosome inactivation and ultimately result in sperm apoptosis. A decrease in H3K4me2 intensity may result in abnormal features in E18.5 embryos derived from aged sires, including craniofacial and skeletal abnormalities, oedema, extra digits, and missing eyes (Siklenka *et al.* 2015, Tatehana *et al.* 2020). Finally, an increase in H3K27me2/3 is linked to gene repression; the exact genes affected are not known but H3K27me2/3 silencing is necessary for cell fate specification (Xie *et al.* 2018, Tatehana *et al.* 2020). There are several challenges in analysing histone modification and paternal age, including reduced histone retention in sperm cells due to protamine replacement (Wiles & Selker 2017), and the lack of standardised techniques to measure histone modifications and localisation (Torres-Flores & Hernández-Hernández 2020).

Sperm DNA methylation is a potential mediator of paternal age effects on offspring, particularly in neurological development and function, and embryo development. Furthermore, APA likely plays a role in retained histone PTMs; however, more studies are needed to validate this. DNA methylation analysis techniques are becoming more methyl-sensitive and genomic-specific, providing greater insight into the affected genes and biological pathways in aged males. The same investment should be made to develop and standardise techniques to analyse histone PTMs in mature sperm cells. Chromatin immunoprecipitation (CHiP) array is used to measure the intensity and localisation of histone PTMs. It requires specific, good-quality antibodies and the sample needs to contain many cells with a high level of the DNA-binding protein of interest, which is difficult to obtain with sperm cells due to their reduced histone retention (Gade & Kalvakolanu 2012). An alternative method to study histone PTMs and their localisation is liquid chromatography followed by tandem mass spectrometry or LC-MS/MS. LC-MS/MS protocols can detect and quantify histone PTMs in a high-throughput manner (Abshiru *et al.* 2020, Zahn *et al.* 2025).

## Paternal age and small noncoding RNAs

Over two decades ago, researchers hypothesised the following: microRNAs (miRNAs) are tissue-specific, are conserved among species, and are likely involved in epigenetic modification of chromatin (Mattick 2003). Shortly after, in 2005, sperm sncRNAs were first identified in the ejaculate of six men with normal fertility (Ostermeier *et al.* 2005). Since then, sperm sncRNAs have been implicated in spermatogenesis, embryo development, and transgenerational inheritance of paternal effects (reviewed in Naveed *et al.* (2025)).

The spermatogenic sncRNA repertoire of mammals is dynamic and determined by specialised extracellular vesicles, called epididymosomes (Belleannée *et al.* 2013). Several studies have found that epididymosomes are responsible for acquisition and loss of different sncRNAs (reviewed in Naveed *et al.* (2025)). The epididymis is comprised of three segments: caput, corpus, and cauda, where the cauda produces mature spermatozoa. At each segment, epididymosomes with different concentrations of small RNAs interact with maturing sperm cells (Belleannée *et al.* 2013).

To date, researchers have established that differential sncRNA expression is linked to paternal age. Most rodent studies observe a correlation between APA and differential sncRNA expression and have linked these changes to reduced sperm capacity and aberrant embryo development (Ma *et al.* 2020, Suvorov *et al.* 2020, Miyahara *et al.* 2023).

### sncRNA expression and reproductive outcomes

In humans, reproductive success is often assessed by a range of reproductive outcomes, such as pregnancy rates, live birth rates, infant health, intergenerational fertility, and IVF/ICSI success (embryo development) (Duffy *et al.* 2020).

Of the few studies on APA-related changes in sperm sncRNAs, most report that the affected RNA profiles are involved in cell development pathways, specifically in post-fertilisation embryo development. Research conducted in human and animal models has used RNA sequencing, microarray techniques, and functional analyses to explore the association between APA and sncRNA profiles (Ma *et al.* 2020, Suvorov *et al.* 2020, Miyahara *et al.* 2023). Table 1 summarises the ages of animals used in the epigenetic studies discussed in this review, relative to the corresponding chronological ages in men. Most studies report that miRNA expression was affected by advanced paternal age. In both C57BL/6J mice (12 vs 20 months) and Wistar rats (65 vs 120 days), miRNA *let-7b-5p* was found to be differentially expressed with age (Suvorov *et al.* 2020, Miyahara *et al.* 2023). Older mice had upregulated *let-7b-5p* expression, which correlated with *Ppp3r1* expression (Miyahara *et al.* 2023). In mice and humans, the protein PPP3R1 enables phosphate-binding activity and is involved in the calcineurin-NFAT signalling cascade (https://www.ncbi.nlm.nih.gov/gene/19058#top, 14th April 2025), (https://www.ncbi.nlm.nih.gov/gene/5534, 14th April 2025). The latter is a signalling pathway required for normal immune response. In mice, *Ppp3r1* is involved in postsynaptic regulation, including postsynaptic modulation of chemical synaptic transmission and neurotransmitter receptor internalisation (https://www.ncbi.nlm.nih.gov/gene/19058#top, 14th April 2025). Human *PPP3R1* is also implicated in Alzheimer’s disease (https://www.ncbi.nlm.nih.gov/gene/5534, 14th April 2025). Functional analysis of differentially expressed miRNAs (DEmiRNAs) in older rats showed that *Ppp3r1* was among the gene targets affected. In the murine model (C57BL/6J), two other sperm DEmiRNAs, mmu-*miR-10a-5p* and mmu-*miR-146a*-*5a*, found in 20-month-old mice were identified as miRNAs that are transmitted from the sperm to the oocyte during fertilisation. These miRNAs could be candidate sperm miRNAs required to ensure successful fertilisation and subsequent offspring health (Miyahara *et al.* 2023). Sperm mitochondrial-related miRNA expression, such as that of nucleic miRNAs, is subject to environmental changes (Tomar *et al.* 2024). One mitochondrial-related miRNA, *miR-151a-5p*, regulates adenosine triphosphate (ATP) production, and overexpression of *miR-151a-5p* is implicated in men with asthenozoospermia (reduced or absent motile sperm in fresh ejaculate) (Zhou *et al.* 2015). Ma *et al.* (2020) identified a mitochondrial-related miRNA, *miR-574*, that was influenced by sperm ageing and is highly conserved between humans and mice. Using the bioinformatic tool RNA22, it was established that *miR-574* directly targets the mitochondrial gene mt-Nd5 in mice When mouse zygotes were microinjected with *miR-574* mimics, a decrease in embryonic development was observed but it was not significant (Ma *et al.* 2020). An overexpression of one miRNA is not enough to generate a phenotypic effect; this is called the paradigm of miRNA switches (reviewed in Flynt & Lai (2008)). The zygotes did, however, show an increase in reactive oxygen species (ROS), which increases the risk of DNA damage and embryonic developmental arrest (Ma *et al.* 2020).

**Table 1:** Estimated ages of men relative to animals used in studies on sperm epigenetics. The table illustrates the approximate ages of men compared to the animals used in studies on the effects of paternal age on sperm epigenetics and the subsequent outcomes on reproductive capacity and offspring health. The relative ages of mice (C57BL/6) to men are based on a survey of studies that used rodent models in biomedical research and how the ages of the animals can be translated to humans (Jackson *et al.* 2017).

Species or strain/age	Equivalent life stage in men	Study
C57BL6/J Rj		Xie *et al.* (2018)
Young: ∼4 months	>20 years	
Old: >21 months	>56 years	
C57BL6/J		Tatehana *et al.* (2020)
Young: 3 months	20 years	
Aged, 12 months	∼38–47 years	
C57BL6/J		Yoshizaki *et al.* (2021)
Young: 3 months	20 years	
Old: >12 months	∼38–69 years	
Wistar rats		Pilsner *et al.* (2021)
65 postnatal days	20–25 years	
120 postnatal days	40–45 years	
C57BL/6		Ma *et al.* (2020)
Young: 6–8 weeks	∼30 years	
Old: >12 months	>38 years	
Wistar rats		Suvorov *et al.* (2020)
65 postnatal days	20–25 years	
120 postnatal days	40–45 years	
Holstein bulls		Wu *et al.* (2020*a*)
10 months	Pre-pubertal	
12 months	Peri-pubertal	
16 months	Post-pubertal	
C57BL/6		Miyahara *et al.* (2023)
3 months	20–30 years	
12 months	38–47 years	
20 months	56–69 years	
C57BL/6N		Guo *et al.* (2021)
Young: 3–4 months	>20 years	
Old: 14–18 months	∼47–56 years	

Conversely, younger male bulls were shown to have differentially expressed sncRNAs. Sperm sncRNAs from bulls aged 10, 12, and 16 months, representing the pre-, peri-, and post-pubertal stages, respectively, were collected. Ten DEmiRNAs that are exclusively paternally expressed were found to be linked to genes involved in post-fertilisation cleavage (Wu *et al.* 2020*a*). The authors used the bioinformatic tool ingenuity pathway analysis (IPA) to link differentially expressed genes to biological pathways. The ten chosen bovine miRNAs targeted genes in several biological pathways including embryonic stem cell pluripotency, thrombin signalling, telomerase signalling, PI3K signalling, iCOS-iCOSL signalling, inhibition of angiogenesis by TSP1, and non-small-cell lung cancer signalling (Wu *et al.* 2020*a*). In a previous study, blastocysts were generated from the sperm of bulls of similar age (Wu *et al.* 2020*b*), and the transcriptomic data collected were used to evaluate the effect of paternal age on the later stages of embryo development. Upstream regulators of differentially expressed genes found in blastocysts at i) 10 vs 16 months and ii) 12 vs 16 months were also targets of three and four miRNAs, respectively. IPA revealed that the downstream targets of these regulators are involved in the following pathways: mitochondrial dysfunction, EIF2 signalling, and sirtuin signalling pathway. Collectively, compared to older bulls, differentially expressed miRNAs in younger bulls affected canonical pathways involved in metabolism, pluripotency, and immune response in both post-fertilisation embryos and blastocysts (Wu *et al.* 2020*a*).

These studies, albeit few, demonstrate how differentially expressed age-related sncRNAs can affect biological pathways, namely those involved in cell death and cellular development. Notably, Miyahara *et al.* (2023) identified pathways affected by the target genes of DEmiRNAs and showed how age-related sncRNAs affected transcription of their target genes. Integrative analysis between hierarchical biological pathways (i.e. transcriptome and epigenome) provides a clearer picture of the aetiology and epidemiology of failed reproductive outcomes related to environmental factors, such as age (Lombardo *et al.* 2022). The next section will discuss how sperm sncRNAs could be potential mediators of paternal age effects on offspring health.

### sncRNA expression: a potential mechanism of paternal age effects

Using a mouse model, Miyahara *et al.* (2023) searched the autism-related gene database, Simons Foundation Autism Research Initiative (SFARI), and identified autism genes that overlapped with the target genes of age-related DEmiRNAs in older and younger mice. Four SFRAI genes (*Oxtr, Gabrb2, Ctnnb1,* and *Grik2*) had significantly different expression levels and were chosen for further analysis. Each gene had a corresponding regulatory sncRNA: mmu*-miR-466j, *mmu*-miR-24-3p, *mmu*-miR-690,* and mmu*-let-7b-5p* (Miyahara *et al.* 2023). A significant negative correlation between mmu*-miR-466j* and the gene *Oxtr*, and between mmu*-miR-24-3p* and the gene *Gabrb2,* was observed, indicating that both miRNAs are negative regulators of the respective genes (Miyahara *et al.* 2023). These genes are orthologous to corresponding genes in humans. *Oxtr* is involved in positive regulation of cold-induced thermogenesis (https://www.ncbi.nlm.nih.gov/gene/18430, 14th April 2025) and is predicted to facilitate protein receptor binding. In humans, *OXTR* encodes the oxytocin receptor (https://www.ncbi.nlm.nih.gov/gene/5021, 14th April 2025). *Gabrb2* is involved in regulating postsynaptic membrane potential and development in the central nervous system (https://www.ncbi.nlm.nih.gov/gene/14401, 14th April 2025) and has a human orthologue, *GABRB2,* with a similar function (https://www.ncbi.nlm.nih.gov/gene/2561, 14th April 2025). A significant positive correlation between mmu*-miR-690* and the gene *Ctnnb1* was observed, demonstrating that mmu*-miR-690* is a positive regulator (Miyahara *et al.* 2023). Murine *Ctnnb1* forms adherens junctions and mediates cell–cell adhesion. It is also a signalling molecule in the canonical Wnt signalling pathway, which mediates cell growth and development (https://www.ncbi.nlm.nih.gov/gene/12387, 14th April 2025). Human *CTNNB1* also forms adherens junctions and acts as an anchor for the cytoskeleton (https://www.ncbi.nlm.nih.gov/gene/1499, 14th April 2025). There was a non-significant correlation between mmu*-let-7b-5p* and *Grik2,* an excitatory neurotransmitter in mice (https://www.ncbi.nlm.nih.gov/gene/14806, 14th April 2025) and humans (*GRIK2*) (https://www.ncbi.nlm.nih.gov/gene/2898, 14th April 2025) (Miyahara *et al.* 2023). These age-related DEmiRNAs target genes that are essential for normal synaptic development and correct growth via cell adhesion. Researchers highlighted that polymorphisms in the human *OXTR* and *GABRB2* genes are linked to ASD. They did note that the full functions of *Oxtr* and *Gabrb2* in brain function are not clear, however, these data demonstrate that paternal age can affect expression of these genes through aberrant miRNA expression, and this can increase the risk of ASD in offspring (Miyahara *et al.* 2023).

Paternal age has also been shown to affect the tsRNA profile of sperm. The intergenerational effects of these age-related tsRNAs were measured in two ways: i) sperm tsRNAs from aged and young F0 mice were injected into preimplantation embryos and gene expression was measured in two-cell embryos and blastocysts; and ii) F1 progeny were generated by injecting zygotes with the same tsRNAs, followed by analysis of gene expression (Guo *et al.* 2021). Both two-cell embryos and blastocysts expressed genes that overlapped with the target genes of differentially expressed tsRNAs. Based on Kyoto Encyclopedia of Genes and Genomes analysis, several of the downregulated genes related to neurological disease, nerve signal transduction, neurotrophin signalling, cholinergic synapses, and other neural signalling pathways (Guo *et al.* 2021). In F1 males, behavioural testing showed that those sired by aged males displayed anxiety-like behaviour. Tissues from the cerebral cortex and hippocampus of F1 males were analysed using RNA sequencing, and a significant difference in gene expression was observed (Guo *et al.* 2021). Together, these results demonstrate that anxiety-like behaviour can be transmitted to offspring via age-altered tsRNAs (Guo *et al.* 2021).

## Conclusion

Parental age is an important factor in fertility. It has long been established that human female fertility begins to decline at the age of 35, and at menopause women stop ovulating (Ishikawa-Yamauchi *et al.* 2024). However, emerging data suggest that male age at conception is also important for reproductive success and that advanced paternal age effects can be transferred to the offspring (Salonia *et al.* 2025). Despite this, the mechanism(s) by which paternal age induces these changes via the ejaculate is not fully understood. Epigenetic mechanisms, such as sperm DNA methylation, are known to be impacted by age. For example, AgeDMRs have been shown to affect genes involved in neurological development (Pilsner *et al.* 2021, Yoshizaki *et al.* 2021, Bernhardt *et al.* 2023). Although only two studies have explored the impact of paternal age on histone posttranslational modifications, they both demonstrated that advanced paternal age can affect spermatogenesis and embryonic development (Xie *et al.* 2018, Tatehana *et al.* 2020). Investigators have now found that small RNAs in mature sperm are not just residual RNAs from the testis, but rather are transcripts that are delivered to epididymal sperm and are important in embryo development and transmission of paternally acquired metabolic and mental disorders (Suvorov *et al.* 2020, Guo *et al.* 2021, Miyahara *et al.* 2023, Naveed *et al.* 2025). There are several constraints to uncovering the mechanistic action of transgenerational inheritance via sncRNAs. Such research requires longitudinal studies, which, in humans, would involve recruiting a multi-generational cohort (Naveed *et al.* 2025). However, animal models can be used to study the effects of paternal age over multiple generations and have the added advantage of being utilised in experiments for understanding causal links between APA, epigenetic mechanisms, and fitness outcomes for offspring and their descendants. Indeed, several of the studies discussed here have used animal models to determine the effect of age on epigenetic mechanisms (Xie *et al.* 2018, Suvorov *et al.* 2020, Tatehana *et al.* 2020, Wu *et al.* 2020*a*, Guo *et al.* 2021, Oluwayiose *et al.* 2021, Pilsner *et al.* 2021, Yoshizaki *et al.* 2021, Bernhardt *et al.* 2023, Miyahara *et al.* 2023, Potabattula *et al.* 2023), but few have used such models to study transgenerational inheritance of paternal effects. Studies on the effect of paternal age on pregnancy outcomes and offspring well-being often adjust for maternal age effects, as older men are likely attempting to conceive with older women. However, as maternal and paternal ages are collinear, separating both parental ages becomes complicated (Halvaei *et al.* 2020). Nevertheless, establishing paternal age effects is important, especially in developed countries where delayed parenthood is becoming more frequent. In conclusion, this review has demonstrated that paternal age is an important factor in sperm epigenetics but also highlights a number of key priorities for further research. Specifically, we have highlighted the likely role of sperm sncRNAs in embryo development and inheritance of paternal age effects. As discussed, this topic is still in infancy and focused and detailed studies in this area are largely lacking. Indeed, the impetus for writing this prospective review was to highlight the need for further work to improve our understanding of sperm-borne sncRNAs and their role in reproductive outcomes and offspring health outcomes.

## Thoughts for the future

The link between paternal age and sperm epigenetics has highlighted the complexity of fertility. Moreover, it has demonstrated that we have a lot to learn about the underlying causes of infertility. But not all hope is lost, as recent advancements in omics techniques have revealed that we can use these to study gamete epigenomics and its effects on the transcriptome, bringing us one step closer to identifying and treating infertility aetiologies. We have shown the presence and functional importance of sperm sncRNAs and their roles in pre-implantation development and postnatal life through to adulthood. We need to gain a better understanding of the mechanistic action of sperm sncRNAs, and how paternal age effects can be transferred to offspring via altered sncRNA profiles. We propose that transgenerational studies in animal models can be used to explore paternal age effects on offspring, as similar studies on paternal diet have been conducted.

## Declaration of interest

The authors declare that there is no conflict of interest that could be perceived as prejudicing the impartiality of the work reported.

## Funding

This research did not receive any specific grant from any funding agency in the public, commercial, or not-for-profit sector.
